# Exploratory study of reactive agility and sprint performance during Ramadan intermittent fasting in male adolescent handball players from a Tunisian club

**DOI:** 10.3389/fphys.2026.1778885

**Published:** 2026-04-17

**Authors:** Mohamed Riadh Bedoui, Mohamed Amine Ltifi, Aymen Bourezgui, Constantin Șufaru, Silviu Ioan Pavel, Anișoara Sandovici, Dan Iulian Alexe, Ridha Aouadi

**Affiliations:** 1 Higher Institute of Sport and Physical Education of Ksar Saîd, Research Laboratory (LR23JS01), “Sport Performance, Health and Society”, University of Manouba, Manouba, Tunisia; 2 Higher Institute of Sport and Physical Education of Gafsa, University of Gafsa, Gafsa, Tunisia; 3 Mining Research Center, Northern Border University, Arar, Saudi Arabia; 4 Department of Physical Education and Sports Performance, “Vasile Alecsandri” University of Bacau, Bacău, Romania; 5 Department of Physical and Occupational Therapy, “Vasile Alecsandri” University of Bacau, Bacău, Romania

**Keywords:** handball, intermittent fasting, Ramadan fasting, reactive agility, sprint performance, U18 athletes

## Abstract

**Background:**

This exploratory study investigated performance changes across the Ramadan period in adolescent U18 male handball players, focusing on reactive agility, linear sprint performance, and pre-planned change-of-direction (Modified Agility Test: MAT) in U18 male handball players. Given the observational design, findings should be interpreted cautiously.

**Methods:**

Thirty adolescent players (mean age 17.5 ± 0.09 years) from a competitive Tunisian club were assessed at five time points: before Ramadan, during weeks 1, 2, and 4, and two weeks post-Ramadan. Tests included 10 m, 20 m, and 30 m sprints, the Modified Agility T-test (MAT), and a Y-shaped reactive agility test (RAT). Data were analyzed using repeated-measures ANOVA.

**Results:**

RAT performance progressively deteriorated across Ramadan and remained impaired two weeks post- Ramadan (p < 0.001). Sprint performance showed transient improvements early in Ramadan, followed by declines, particularly over 20–30 m. MAT performance remained relatively stable across all time points. Body mass and body mass index decreased significantly during Ramadan (p < 0.001) and returned close to baseline post-Ramadan.

**Conclusion:**

This exploratory study observed selective performance changes across the Ramadan period, with RAT declining most consistently, sprint performance showing transient changes, and MAT remaining relatively stable despite statistically significant differences. These findings may reflect the combined influence of physiological and contextual factors. However, due to the observational design and absence of a control group, causality cannot be established and results should be interpreted cautiously.

## 1 Introduction

Handball is a high-intensity, intermittent team sport characterized by frequent alternations between low and high-intensity activity phases. It requires repeated explosive actions such as sprinting, jumping, and rapid changes of direction, often performed under fatigue and tactical pressure (Karcher and Buchheit, 2014). Success at the elite level depends not only on well-developed physical attributes but also on the integration of perceptual-cognitive skills, particularly rapid decision-making and situational awareness (Hinz et al., 2022).

For Muslim athletes, Ramadan intermittent fasting (RIF) introduces significant challenges to training and competition (Ghaffar et al., 2025). During Ramadan, abstention from food and fluids between dawn and sunset alters hydration, circadian rhythms, nutrient availability, and sleep patterns, all of which can affect recovery and competition readiness (Abaïdia et al., 2020). While overall performance decrements are often modest, high-intensity qualities such as sprinting, repeated efforts, and agility appear especially vulnerable, with impairments most evident later in the day (Bougrine et al., 2023a). Nevertheless, results remain inconsistent: while elite athletes with structured routines may show negligible decrements (Chaouachi et al., 2009b; Chaouachi et al., 2012), adolescent players and sub-elite populations often demonstrate significant declines (Özbay et al., 2024). These discrepancies highlight the role of contextual moderators, including training time, nutritional strategies (ManChado et al., 2013), and adaptations such as delaying the pre-dawn meal (Bougrine et al., 2023b) or caffeine supplementation (Bougrine et al., 2023a).

Unlike pre-planned change-of-direction (MAT), reactive agility requires perceptual input, anticipation, and rapid execution in response to unpredictable stimuli (Chaouachi et al., 2009a; Koo and Li, 2016; Sammoud et al., 2021). In handball, reactive agility is a critical determinant of both offensive and defensive effectiveness (Spasic et al., 2015). Evidence indicates that professional handball players outperform athletes from other invasion sports in reactive agility (Harder-Lauridsen et al., 2017; Gudden et al., 2021). Among adolescent players, fundamental motor skills such as sprinting and jumping are strong predictors of reactive agility (França et al., 2022; Pavlinovic et al., 2022).

To our knowledge, no previous studies have specifically examined how intermittent fasting affects reactive agility in adolescent players. Accordingly, this exploratory study aimed to investigate the effects of RIF on linear sprint performance, reactive agility, and pre-planned change-of-direction ability in male adolescent U18 handball players. Given the exploratory scope of this investigation, findings should be interpreted cautiously and serve as a basis for future controlled trials. We hypothesized that RIF would impair all performance measures, with the greatest decrements in reactive agility due to its added cognitive demands. By addressing this underexplored intersection of fasting, adolescent players’ development, and cognitively demanding performance outcomes, the study aims to provide practical guidance for coaches on scheduling, nutrition, and recovery during Ramadan fasting.

## 2 Methods

### 2.1 Participants

Thirty male U18 handball players from a single competitive handball club in Tunisia (n = 30) voluntarily participated in this study. Baseline anthropometrics are summarized in **Table 1**. Inclusion criteria were: (1) active club registration with regular training participation (≥4 sessions per week plus one official match), (2) full observance of RIF (no food or fluid intake from dawn to sunset) throughout the entire month, and (3) absence of any musculoskeletal injury preventing maximal effort within the 3 months data collection. Players and their parents/guardians were informed verbally and in writing about the study aims and procedures. Written assent was obtained from players, and written consent from their parents, prior to enrollment. The study was conducted during Ramadan of 2025 in Tunisia.

**Table 1:** Age and anthropometric characteristics of U18 handball players (mean ± SEM; n = 30).

Variables	Mean ± SEM
Age (years)	17.50 ± 0.09
Body mass (kg)	80.67 ± 2.54
Height (cm)	181.63 ± 1.26
Body Mass Index (kg·m⁻²)	24.34 ± 0.59

### 2.2 Anthropometric measurements

The following anthropometric parameters were measured: body mass, height, and body mass index (BMI) (**Table 1**). Measurements were performed on the same day following a standardized sequence. Body mass was recorded to the nearest 0.1 kg using an electronic scale (Seca, Hamburg, Germany), with participants standing barefoot and wearing light clothing. Height was measured barefoot using a wall-mounted stadiometer and a sitting height table (GPM–Swiss Made). Body composition (fat vs. lean mass) and hydration status were not assessed. Participants were instructed to follow their usual Ramadan hydration routines, but no objective markers of hydration (e.g., urine specific gravity or plasma osmolality) were collected.

### 2.3 Study design and testing schedule

**Figure 1:**
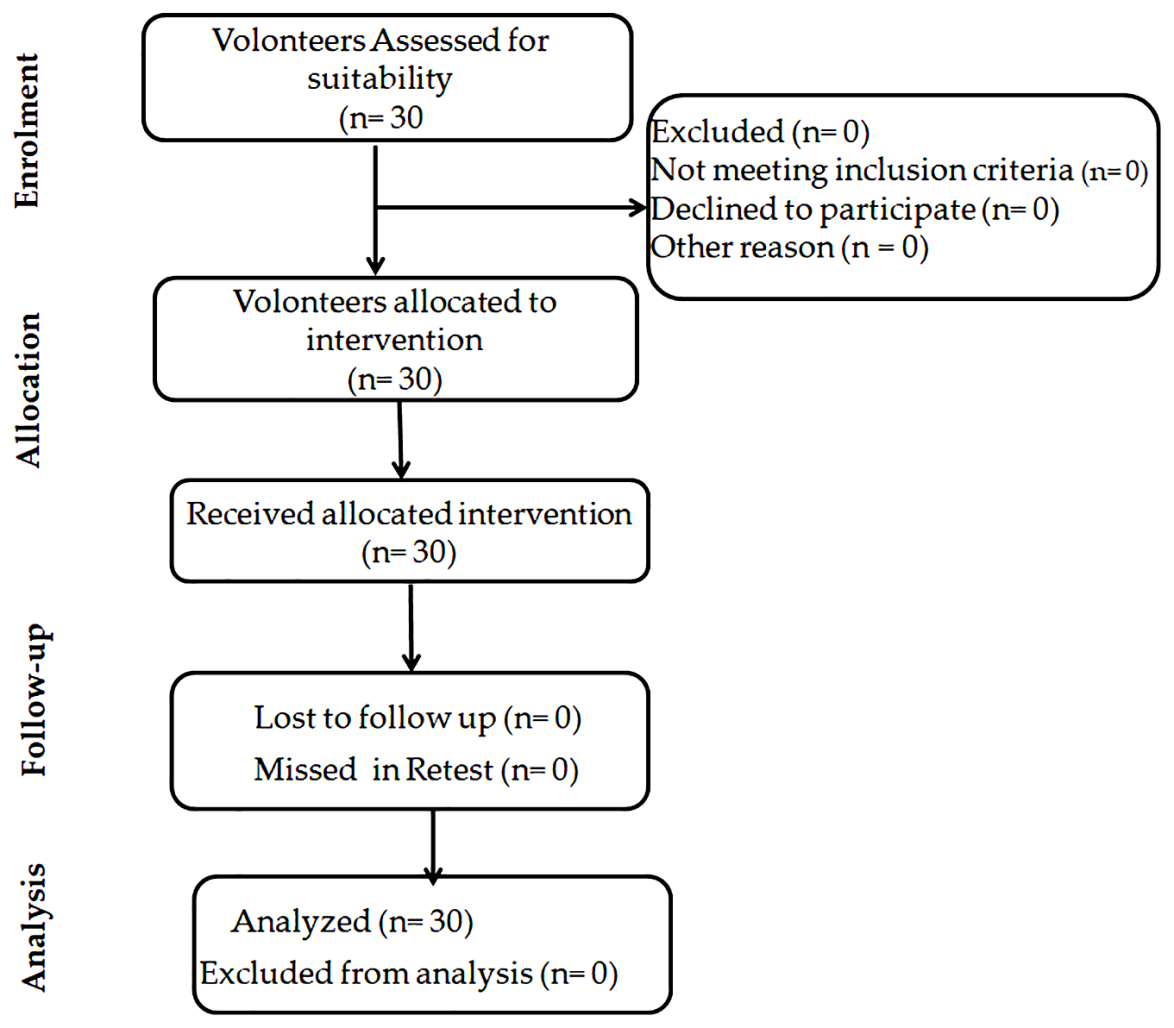
Schematic diagram of the study design illustrating the flowchart of participant recruitment, allocation, follow-up, and analysis in the intervention study (n = 30).

The experimental design is presented in **Figure 1**. The selection of the testing time points was guided by the methodological framework (Fekih et al., 2020), which emphasized the importance of monitoring performance across key phases of Ramadan.

**Figure 2:**
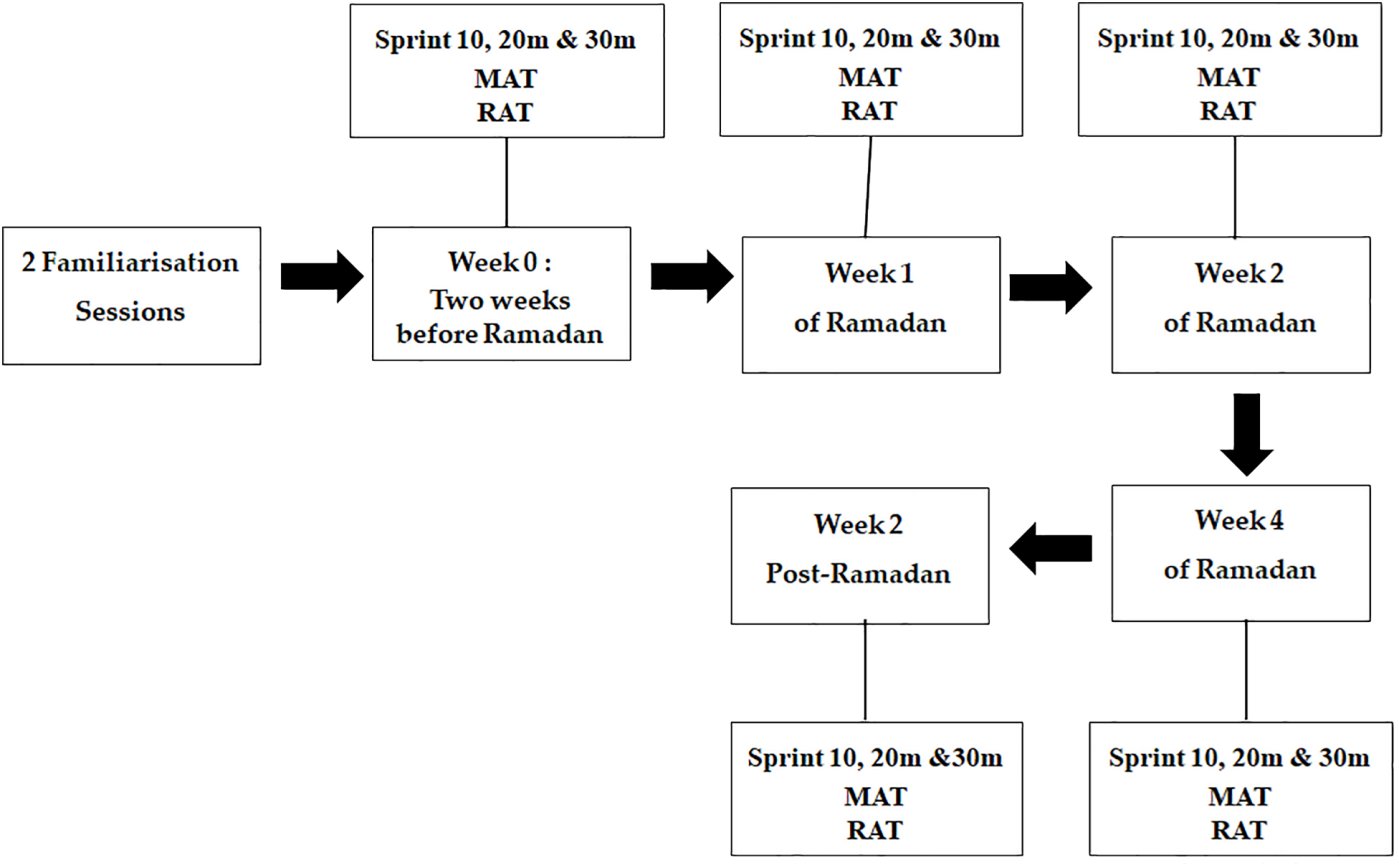
Schematic diagram of the study design showing the five testing time points (Week 0, Week 1, Week 2, Week 4, and Week 2 Post-Ramadan) and the main performance assessments (sprint test, Modified Agility T-test, and Reactive Agility Test). MAT, Modified Agility Test; RAT, Reactive Agility Test.

This study employed a repeated-measures observational design with five time points to evaluate the effects of RIF: T1 (baseline, 2 weeks before Ramadan), T2 (week-1), T3 (week-2), T4 (week-4), and T5 (follow-up, 2 weeks post-Ramadan) (**Figure 2**).

Training load and match schedules were monitored throughout the study period. During Ramadan, training sessions were maintained in terms of timing and intensity. Participants followed a regular training schedule consisting of approximately 4 training sessions per week and one official match. Training sessions were typically of moderate-to-high intensity and included technical, tactical, and physical components. During Ramadan, training sessions were generally scheduled in the late afternoon or evening (i.e., closer to Iftar), in line with common practice, but the overall training content and volume were maintained by the coaching staff. Throughout the study period, participants followed a regular training schedule consisting of approximately 4 training sessions per week and one official match. No major modifications in training structure were reported during the study period. To minimize the potential influence of acute fatigue, all testing sessions were conducted on days without official matches and were separated from intense training sessions by at least 48 hours. All testing sessions were conducted in the team’s indoor sports hall to minimize environmental variability. Sessions were scheduled in the late afternoon, approximately 90 ± 15min before local sunset (Iftar), ensuring that all participants were tested under comparable fasting conditions, when dehydration and hypoglycemia are likely to peak. Participants were also instructed to avoid strenuous exercise in the 48 hours preceding testing, as described above.

Participants completed two familiarization sessions, separated by at least 48 hours, prior to baseline testing to reduce learning effects. Familiarization sessions included the full testing battery. No participants were excluded at any time point, and all analyses were performed on the full sample (n = 30). The participants provided their written informed consent to participate in this study.

### 2.4 Pre-test standardization and hydration

Participants were instructed to: (a) maintain their usual Ramadan sleep and nutrition routines, (b) refrain from strenuous exercise for 24 h before testing, and (c) fully adhere to Ramadan fasting rules (no food or fluid intake from dawn to sunset). Participants with acute illness, injury, or who did not pass fasting verification were rescheduled or excluded. Immediately before each testing session during Ramadan, players were interviewed individually in a private setting using a standardized two-item script: (1) “Do you confirm that you are fasting for Ramadan today (no food or fluid intake since dawn)?” (Yes/No); (2) “Do you confirm that your last pre-dawn meal/drink ended before today’s suhoor deadline time?” (Yes/No). Participants were cleared to test only if they answered “Yes” to both items; any “No” or “Unsure” prompted same-day postponement and rescheduling within the same time point (T1 to T5). Although participants were asked to maintain their habitual sleep and hydration routines during Ramadan, we did not objectively measure sleep duration, sleep quality, or detailed post-Iftar nutritional intake. Therefore, variations in circadian rhythm, sleep timing, and meal composition may have contributed to the observed performance changes.

### 2.5 Testing procedures and warm-up

**Table 2** summarizes the standardized testing procedures and warm-up protocol used to ensure consistency and reliability across all testing sessions during Ramadan.

**Table 2:** Testing procedures and warm-up protocol.

Component	Description	Rationale/purpose
Warm-Up Protocol	Total duration: ~23 minutes 1. General warm-up (8 min): Standardized jogging. 2. Specific warm-up (15 min): Sport-specific exercises and mobility drills.	To prepare participants physiologically and specifically for the subsequent physical tests. All sessions were supervised by the same researcher to ensure consistency.
Testing Sequence	Tests were performed in the following fixed order: 1. Linear Sprint (10m, 20m and 30m) 2. Modified Agility T-test (Pre-planned Change of Direction) 3. Y-shaped Reactive Agility Test.	To minimize fatigue carry-over and maximize test reliability. The sequence progresses from less fatiguing and less cognitively demanding tasks to more complex, reactive tasks.
Rest Periods	• Inter-Test Type: A passive rest of 10 minutes was provided between each test type (e.g., between Sprint and T-test). • Intra-Test Recovery: Standardized recovery periods were implemented within each test.	To ensure adequate recovery between different performance measures, preventing fatigue from influencing subsequent test results.

### 2.6 Measurement details

#### 2.6.1 Speed tests

Sprint performance was assessed over 10 m, 20 m, and 30 m using Microgate photoelectric timing gates (Microgate, Bolzano, Italy) (Sammoud et al., 2021). Participants started 0.5 m behind the line and sprinted maximally upon the “ready–go” cue, and photocells were positioned 0.4 m above the ground (Spasic et al., 2015). To reduce fatigue and improve ecological validity, 10 m, 20 m, and 30 m sprint times were recorded within a single maximal sprint using split timing gates. Each participant performed two trials, separated by 3 minutes of passive recovery, with the best trial retained for analysis. Footwear and surface were standardized across all testing sessions (indoor court; same pair of court shoes for each athlete).

#### 2.6.2 Modified Agility T-Test

**Figure 3:**
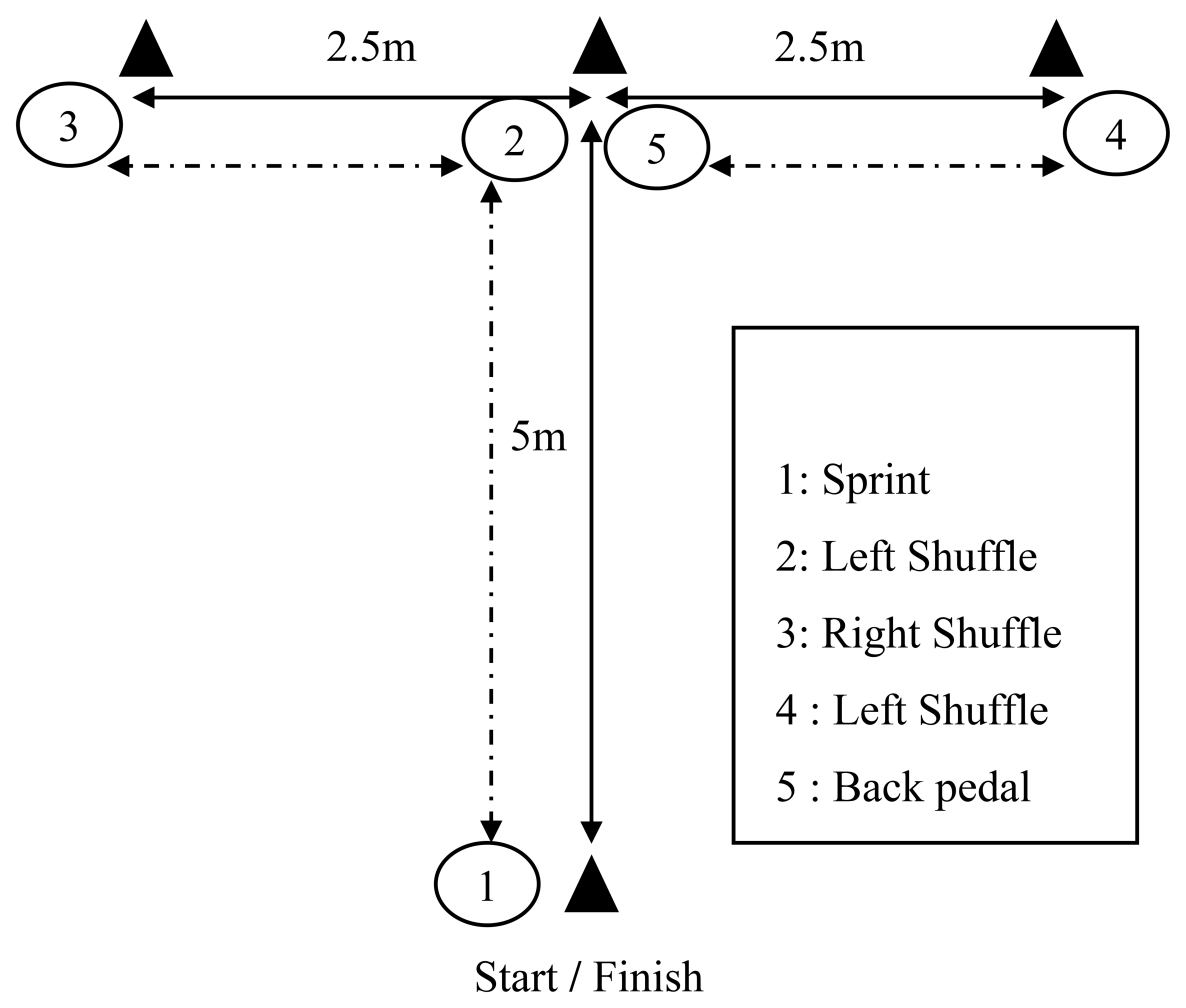
Diagram depicting the movement sequence of the MAT, adapted from a previously validated protocol (Sassi et al., 2009). The test includes: (1) forward sprint, (2) lateral shuffle to the left, (3) lateral shuffle to the right, (4) return to the center cone, and (5) final sprint.

The MAT was employed to evaluate change-of-direction speed, incorporating forward acceleration, lateral movements to both sides, and backward displacement (Sassi et al., 2009). Performance times were obtained using a photocell timing system (Microgate, Bolzano, Italy). Each player performed two trials, separated by at least 3 minutes of rest, and the best time was retained for subsequent analysis. Before starting, participants positioned both feet on a reference line placed 30 cm behind the timing gate (Mathisen and Danielsen, 2014). The protocol consisted of the following sequence: (1) a 5-m forward sprint to cone 2, followed by touching its base with the right hand (**Figure 3**); (2) a 2.5-m lateral shuffle to the left toward cone 3, maintaining proper footwork (no crossover), and touching the cone with the left hand; (3) a 5-m lateral shuffle to the right toward cone 4 with a right-hand touch; (4) a return to cone 2 over 2.5 m to touch its base; and (5) a final sprint through the finish line as fast as possible [**Figure 3** (Sassi et al., 2009)]. Footwork rules and penalties for incorrect technique were standardized across all sessions. Testing conducted on the same indoor surface with the same footwear as used for the sprints assessments.

#### 2.6.3 Y-shaped Reactive Agility Test

**Figure 4:**
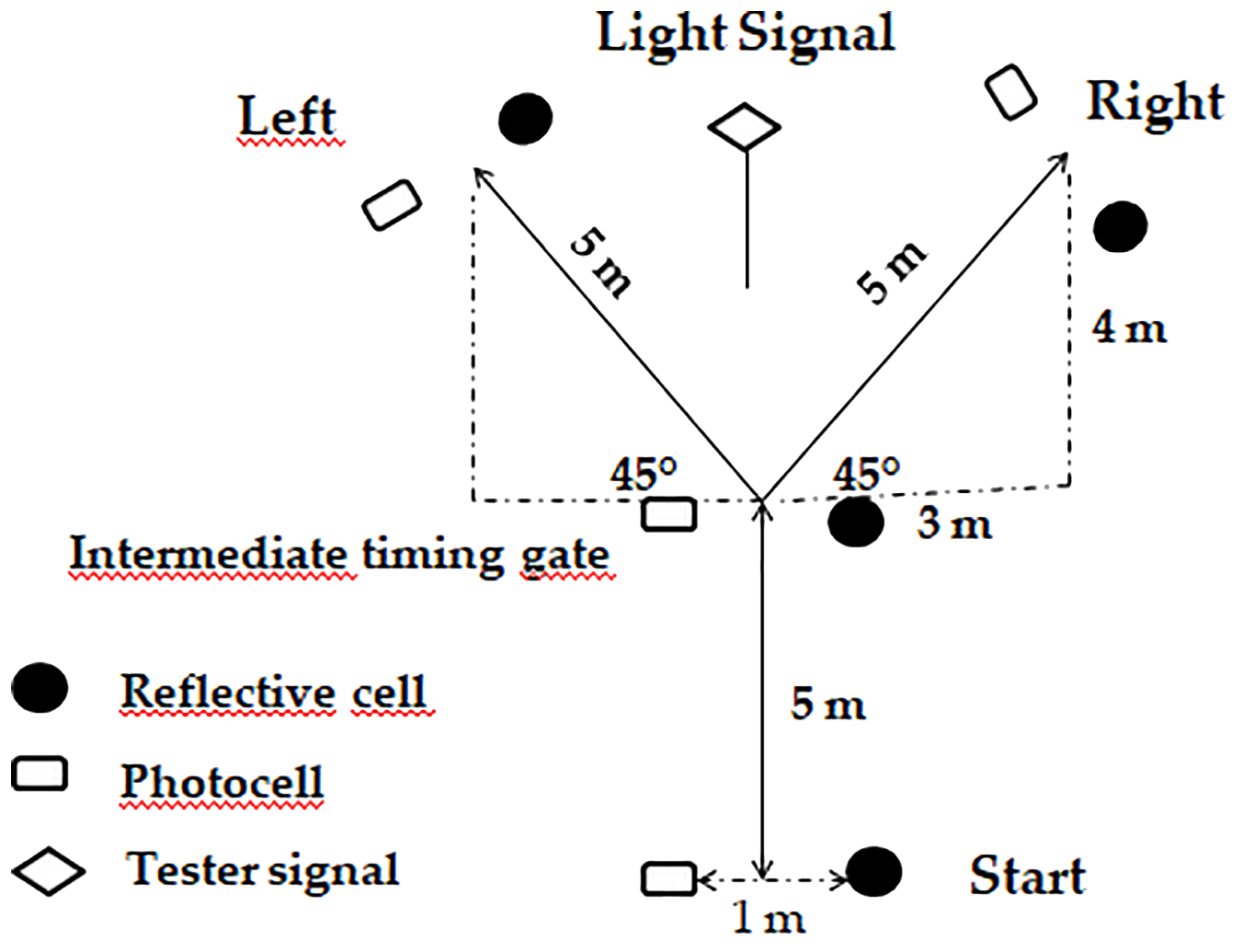
Schematic representation of the RAT setup and pathway, adapted from a previously validated protocol (Chtourou et al., 2011). Participants sprint forward and respond to a visual stimulus indicating the direction (left or right) after crossing the midpoint.

A schematic representation of the RAT setup is provided in **Figure 4**. Each sprint began from a starting line positioned 30 cm behind the first timing gate. Sprint performance was assessed using photoelectric timing gates (Microgate, Bolzano, Italy). In the middle of the course, the timing system was installed outside foam barriers separated by 1 m. Exit gates were positioned on both the left and right sides of the track, aligned perpendicularly to the running path. Sprint times were transmitted wirelessly to a handheld device for further processing. The timing system also determined the direction to be taken after the initial 5 m sprint. Once the midpoint beam was crossed, a light signal appeared on one of the exit gates (left or right), indicating the direction to follow. Participants had to respond to this cue and sprint as fast as possible toward the illuminated gate (**Figure 4**). The time delay between breaking the midpoint beam and the light stimulus was approximately 40–45 ms. To avoid anticipation, participants were instructed not to guess the direction, and the researcher monitored adherence by observing their execution and comparing reactive versus pre-planned sprint times (Chtourou et al., 2011).

### 2.7 Statistical analysis

Statistical procedures were performed in IBM SPSS Statistics v.27 (IBM Corp., Armonk, NY, USA). Data are expressed as mean ± SEM. Normality was assessed using the Shapiro–Wilk test. Sphericity was evaluated with Mauchly’s test, with Greenhouse–Geisser corrections applied when violated. The primary analysis was a one-way repeated-measures ANOVA with Time (five levels: Week 0 to Week 2 Post-Ramadan) as the within-subject factor. Significant main effects were followed by Bonferroni-adjusted pairwise comparisons across the five time points. The consistency of the tests across days was examined using the intraclass correlation coefficient (ICC), calculated from the best performance recorded each day. An ICC in the range of 0.70–0.80 was regarded as doubtful, whereas values above 0.90 were interpreted as demonstrating excellent reliability (Pavlinovic et al., 2022). Reliability indices (ICC and CV%) were reported for sprint and MAT and RAT. Effect size indices (partial eta squared) were instead used to quantify the magnitude of changes over time. Effect sizes are reported as partial eta squared (ηp²) and Cohen’s d for repeated measures which was calculated using the approach proposed by Morris and DeShon, based on the standard deviation of the difference scores, to provide an appropriate estimate of within-subject effect size. The Cohen’s d values were interpreted according to conventional thresholds: small (d = 0.2 to 0.49), medium (d = 0.5 to 0.79), and large (d ≥ 0.8). Effect sizes were interpreted using conventional benchmarks; however, their practical relevance was considered in light of the small absolute changes and the reliability of the measurements.

An *a priori* power analysis was conducted using G*Power (v3.1.9.x) for a within-subject repeated-measures design with five levels. Parameters were set as follows: medium effect size (F = 0.25), significance level (α = 0.05), statistical power (1−β = 0.80), and assumed within-subject correlation = 0.50. This approach aligns with prior methodology (Bougrine et al., 2023b), who employed G*Power with comparable parameters to determine sample size in agility-focused studies during Ramadan fasting. The analysis indicated a minimum required sample of 21 participants; to accommodate potential attrition, 30 participants were recruited. All analyses were two-tailed, with significance set at p < 0.05.

## 3 Results

The anthropometric characteristics of the experimental handball players are presented in **Table 3**. Body mass and BMI showed significant reductions during Ramadan (p < 0.001; ηp²= 0.969 and 0.966 respectively for body mass and Body Mass Index: BMI).

**Table 3:** Body Mass and Body Mass Index over time (mean ± SEM) (n = 30).

Parameter	Week 0	Week 1	Week 2	Week 4	Week 2 Post-Ramadan	ηp²
BM (kg)	80.67±2.54	78.71±2.52^a^	77.69±2.53 ^a^	76.54±2.51 ^a^	77.67± 2.50 ^a^	0.969
BMI (kg/m^2^)	24.34±0.59	23.74±0.59 ^a^	23.43±0.59 ^a^	23.08±0.58 ^a^	23.43±0.58 ^a^	0.966

^a^ significantly different (p < 0.001) compared to Week 0; BM, Body Mass; BMI, Body Mass Index; ηp², Partial Eta Squared (effect size).

### 3.1 Anthropometric characteristics

### 3.2 Sprint performance

Sprint outcomes are summarized in **Table 4**. During Ramadan, sprint performance showed a transient improvement followed by attenuation. At Week 2 and week 4, 20 m (3.297 ± 0.026 s) and 30 m (4.596 ± 0.036 s) times were faster than baseline (p < 0.01). Two weeks post-Ramadan, sprint times partially recovered but remained slower than baseline (p < 0.01). Sprint 10 m was largely stable from Week 0 to Week 4, but was slower two weeks post-Ramadan compared with all Ramadan time points (p < 0.01).

**Table 4:** Sprint, Modified Agility Test (MAT) and Reactive Agility Test (RAT) performances across measurement periods during and after Ramadan (mean ± SEM, ICC, CV%, and partial eta squared).

Parameter	Week 0	Week 1	Week 2	Week 4	Week 2 Post-Ramadan	ICC	CV (%)	ηp²	Cohen’s d
Sprint 10m (sec)	1.921 ± 0.018 ^b^	1.924 ± 0.018 ^b^	1.930 ± 0.020 ^b^	1.934 ± 0.018 ^abd^	1.961 ± 0.018 ^a^	0.81-0.99	1.98-3.96	0.292	0.27
Sprint 20m (sec)	3.321± 0.026	3.326± 0.026	3.297± 0.026 ^ad^	3.337± 0.027 ^ad^	3.368± 0.026 ^ad^	0.79-0.80	2.03-2.06	0.884	0.21
Sprint 30m (sec)	4.630± 0.037 ^bc^	4.637± 0.037 ^bc^	4.596± 0.036 ^abc^	4.654± 0.037 ^ab^	4.690± 0.037 ^ac^	0.86-0.87	1.71-1.73	0.862	0.59
MAT (sec)	9.314 ± 0.135 ^bc^	9.337 ± 0.134^b^	9.244 ± 0.133^adc^	9.367 ± 0.136 ^ab^	9.383 ± 0.134 ^abcd^	0.97-0.98	1.46-1.47	0.828	0.51
RAT (sec)									
T1 (sec)	1.279 ± 0.019^bcd^	1.284 ± 0.019^abc^	1.271 ± 0.018^$ abd^	1.296 ± 0.019^# abd^	1.322± 0.019^acd^	0.98-0.99	0.96-4.98	0.895	0.86
T2 (sec)	1.600 ± 0.021^bc^	1.605 ± 0.21^bc^	1.587 ± 0.020 ^abcd^	1.616 ± 0.021 ^abd^	1.643± 0.021 ^acd^	0.98-0.99	0.40-4.52	0.889	0.69
Total Time (sec)	2.813± 0.050 ^b^	2.889± 0.034^bc^	2.858± 0.033^bcd^	2.912± 0.034 ^bd^	2.964± 0.034 ^acd^	0.97-0.99	0.67-4.96	0.206	1.27

^a:^significantly different (^a^p < 0.05; ^a^p < 0.001) compared to Week 0; ^b^significantly different (p < 0.001) compared to Week 2-Post- Ramadan; ^c^significantly different (^c^p < 0.05; ^c^p < 0.001) compared to Week 4; ^d^significantly different (^d^p < 0.05; ^d^p < 0.001) compared to Week 1.

ICC and CV% values are presented as ranges across the different assessment time points (Week-0 to Post-Ramadan) for each variable.

### 3.3 MAT performance

MAT performance showed a significant increase in completion time starting from Week 2 of Ramadan (p < 0.01), which persisted through Week 4 (p < 0.001). Notably, this impairment remained evident even two weeks post-Ramadan (p < 0.001). Agility performance (**Table 4**) followed a similar pattern. The MAT time increased from 9.314 ± 0.135 s at Week 0 to 9.367 ± 0.136 s at Week 4 (p < 0.01), indicating a statistically significant increase in completion time; however, the absolute difference was small (~0.05 s), suggesting limited practical relevance. Similarly, the RAT showed significantly slower responses, with total time rising from 2.813 ± 0.050 s at baseline to 2.912 ± 0.034 s at Week 4 (p < 0.001). Two weeks after Ramadan, agility performance remained impaired, as reactive agility total time was still elevated compared with Week 0 (2.964 ± 0.034 s, p < 0.001).

### 3.4 Reliability of sprint and agility tests

Reliability indices of sprint, MAT and RAT tests across all periods are summarized in **Table 4**. The ICC indicated good-to-excellent reliability, ranging from 0.78 (95% CI: 0.56–0.90) for the 20 m sprint at Week 2 to 0.97 (95% CI: 0.94–0.99) for the MAT across all weeks. Sprint tests consistently demonstrated ICC values between 0.79 and 0.86, whereas the agility test maintained excellent reliability (ICC = 0.97) throughout the study. The coefficients of variation (CV%) remained low (1.72 to 2.06%), ranging from further confirming stable measurement consistency during Ramadan and post-Ramadan.

Within-session and between-session reliability were computed using ICC and CV%. The RAT demonstrated acceptable to good reliability, with ICC values consistently exceeding 0.70 and CV% values remaining below 5%, indicating satisfactory within-subject consistency.

## 4 Discussion

This exploratory longitudinal study provides novel evidence regarding the effects of RIF on distinct performance domains (linear sprinting, MAT, and RAT) in U18 players. While previous studies reported mixed effects of RIF on physical performance (Zarrouk et al., 2016; Bougrine et al., 2024; Trabelsi et al., 2024), our findings confirm that neuromuscular and perceptual–cognitive qualities do not respond uniformly to fasting. Reactive agility deteriorated progressively and remained impaired post-Ramadan, whereas sprinting showed transient gains before declining.

The persistent decline in reactive agility from Week 2 onward reinforces that tasks requiring rapid perception–action coupling are more vulnerable to physiological stressors than pre-planned movements. Unlike MAT, reactive agility integrates decision-making, visual processing, attention, and rapid motor responses (Broodryk et al., 2025; Horníková et al., 2025). Mild dehydration, sleep disturbances, and reduced carbohydrate availability, all common during Ramadan, are known to impair vigilance and neuromotor accuracy and may have contributed to the greater susceptibility of RAT, although this remains speculative in the absence of direct measurements (Trabelsi et al., 2022; Khemila et al., 2023; Francisco et al., 2024).

These results are consistent with reports of reduced psychomotor processing speed, vigilance, and mental rotation during Ramadan in adolescents (Bougrine et al., 2024), with deficits accumulating as circadian disruption increases (Trabelsi et al., 2022). Because reactive agility relies heavily on cortical processing, cognitive fatigue and altered neuroenergetic states may substantially contribute to these reductions (Badin et al., 2016). Evidence from youth soccer further emphasizes the interdependence of cognitive and physical skills (Ltifi et al., 2025), suggesting that cognitive load during fasting could indirectly modulate MAT.

Although some studies indicate stable simple and choice reaction times during Ramadan in trained athletes (Zarrouk et al., 2016), others report progressive impairments extending post-Ramadan (Zarrouk et al., 2016; Bougrine et al., 2024). This duality may explain why RAT, which requires perception–action integration, declines more sharply than basic neuromuscular tasks in the present exploratory study.

Sprint performance showed slight early improvements before deteriorating later, possibly reflecting a short-term taper effect from reduced training load (Abaïdia et al., 2020; Özbay et al., 2024). As fasting progressed, reduced glycogen availability, increased perceived exertion, and sleep disruption could plausibly interact with the observed performance decrements (Rockwell et al., 2003; Murray and Rosenbloom, 2018; Triki et al., 2024). Persistent decrements post-Ramadan suggest that neuromuscular recovery may lag behind metabolic normalization, supporting recommendations for structured re-acclimatization (Trabelsi et al., 2024).

MAT performance remained stable, indicating that pre-planned mechanical tasks are less affected by fasting-induced cognitive or perceptual strain. MAT depends mainly on strength, stability, and biomechanical efficiency (Fekih et al., 2020; França et al., 2022), qualities relatively preserved under moderate dehydration and reduced energy intake.

Although MAT performance showed statistically significant changes over time, the magnitude of these differences was small and likely of limited practical significance. The observed increase in completion time (~0.05 s) remains minimal and close to the measurement variability (CV%), suggesting that these changes may not reflect meaningful alterations in performance. Although the effect size (ηp²) was large, this likely reflects the repeated-measures design and low within-subject variability. In contrast, the small Cohen’s d values and minimal absolute differences suggest limited practical significance.

In contrast to RAT, which demonstrated larger and more consistent impairments across the Ramadan period, MAT appears relatively stable when interpreted from a practical perspective. This supports the notion that pre-planned change-of-direction tasks are less sensitive to the combined physiological and contextual constraints experienced during this period. MAT primarily depends on neuromuscular and biomechanical capacities, such as strength, coordination, and movement efficiency, which may be relatively preserved despite fluctuations in hydration status, energy intake, and circadian rhythm.

This interpretation aligns with previous evidence showing limited shared variance between MAT and RAT (Spasic et al., 2015) and supports the distinction between their underlying determinants (Khemila et al., 2023; Broodryk et al., 2025). In contrast to RAT, which requires rapid perception–action coupling and decision-making under time pressure, MAT primarily reflects mechanical execution under predictable conditions.

Although the greater impairment observed in RAT compared with MAT performance is interpreted in relation to higher perceptual–cognitive demands, it should be acknowledged that no direct measures of cognitive function or mental fatigue (e.g., reaction time tasks, vigilance tests, or perceived cognitive exertion scales) were included. Consequently, the role of mental fatigue cannot be confirmed directly and remains an inference based on task characteristics rather than explicit cognitive assessment.

Nevertheless, the distinction between RAT and MAT is well established in the literature, with RAT involving perceptual–cognitive processing and rapid decision-making, while pre-planned tasks primarily reflect mechanical and neuromuscular capacities (Spasic et al., 2015; Pavlinovic et al., 2022; Horníková et al., 2025), with RAT requiring stimulus perception, decision-making, and rapid response selection, whereas MAT primarily reflects mechanical and neuromuscular execution under predictable conditions. The divergent temporal patterns observed in the present study—progressive deterioration in RAT alongside relative stability of MAT support the notion that fasting-related stressors may disproportionately affect tasks with higher perceptual–cognitive load. Future studies should integrate objective cognitive and psychomotor measures alongside RAT testing to directly quantify mental fatigue and better clarify underlying mechanisms.

Systematic reviews also report decrements in repeated-sprint ability and intermittent performance during Ramadan (Chtourou et al., 2011; Abaïdia et al., 2020; Trabelsi et al., 2024; Ltifi et al., 2025), consistent with our progressive sprint and MAT impairments. The excellent reliability of sprint and MAT across Ramadan underscores their stability. Although RAT inherently involves greater cognitive and perceptual variability, the acceptable reliability indices observed in the present study support the robustness of the RAT outcomes.

The persistent post-Ramadan deficits observed here further suggest that neuromuscular and cognitive recovery may extend beyond the fasting period, emphasizing the need for continued monitoring and gradual reintroduction of high perceptual and cognitive loads. The post-Ramadan study duration was limited to two weeks, and RAT remained impaired at this point. Future studies should include longer monitoring periods to fully capture the time course of neuromuscular and cognitive recovery after fasting. Future studies should include assessments both before and after Iftar to better understand performance variations throughout the day.

### 4.1 Practical implications

The differentiated effects across performance domains underscore the need for sport-specific planning during Ramadan. Cognitively demanding sessions, particularly RAT drills, should be scheduled after Iftar, while strength-, stability-, or MAT-focused sessions may be performed in a fasted state. Nutritional strategies, including carbohydrate-rich Suhoor and adequate protein at Iftar, can help sustain performance (Spasic et al., 2015; Murray and Rosenbloom, 2018; Bougrine et al., 2023b). A structured post-Ramadan re-acclimatization period is recommended, with gradual increases in training intensity and reintroduction of perceptual–cognitive drills after foundational neuromuscular qualities stabilize. Staged training in the fed state may support recovery, especially considering persistent reductions in sleep quality, hydration, and carbohydrate intake (Trabelsi et al., 2024; Triki et al., 2024).

Finally, the present findings should be interpreted within the specific developmental and sporting context of U18 players. adolescent players may respond differently to fasting compared with adults or elite professionals due to ongoing maturation and differences in training structure. Moreover, potential sex-specific physiological and hormonal responses to fasting, particularly in female athletes, warrant caution when extrapolating these findings beyond the studied population.

### 4.2 Limitations

This study has several limitations that should be acknowledged. The sample size (n = 30) may have limited the detection of very small effects, and the cohort of U18 players from a single Tunisian club may reduce the generalizability of the findings to other populations, sports, or competitive levels. The post-Ramadan assessment period was relatively short, and longer-term adaptations remain unknown. Furthermore, the absence of a non-fasting control group limits the ability to isolate the specific effects of Ramadan from seasonal or training-related factors.

In addition, several important physiological and behavioral variables were not directly measured, including body composition, hydration status, sleep patterns, circadian rhythms, and cognitive function. Consequently, any mechanistic interpretations regarding their impact on performance remain speculative. These factors may have contributed individually or interacted in complex ways to influence performance outcomes; however, their relative contributions cannot be determined within the present design. Future studies should include non-fasting or fed-state control groups, extended observation periods, multi-center samples, and objective assessments of these variables to better elucidate the underlying mechanisms and strengthen external validity.

## 5 Conclusion

In trained male handball players assessed repeatedly before Iftar, this exploratory study observed selective performance alterations across Ramadan, with RAT showing the most consistent and persistent decline even into the post-fasting period. Body mass and BMI decreased, suggesting reduced energy availability, while sprint performance showed early gains that later diminished and change-of-direction speed remained largely stable. Nevertheless, the findings provide preliminary guidance for coaches and researchers and highlight the need for future controlled trials to clarify the mechanisms underlying performance changes during Ramadan and to optimize training strategies for U18 players.

## Data Availability

The original contributions presented in the study are included in the article/supplementary material. Further inquiries can be directed to the corresponding author.
